# Clinical and histopathological manifestations of snus use in Germany: parallels to betel nut–related oral submucous fibrosis in Asia

**DOI:** 10.1007/s00428-026-04414-4

**Published:** 2026-01-23

**Authors:** Lukas Greber, Sven Otto, Philipp Poxleitner, Thierbach René, Stephan Ihrler

**Affiliations:** 1 Department of Oral and Maxillofacial Surgery, Ludwig-Maximilians-Universität Munich, Munich, Germany; 2 Department of Oral and Plastic Maxillofacial Surgery, Military Hospital Ulm, Oberer Eselsberg 40, Ulm, 89081 Germany; 3 Branch of Dentistry, Medical Service of the German Armed Forces, Support Command Bundeswehr, Bonn, Germany; 4 DERMPATH Munich, Munich, Germany

**Keywords:** Snus, Oral mucosal changes, Submucous fibrosis, OSMF, Histopathological analysis, Gingival recession, Erosions, Tooth discoloration

## Abstract

While oral submucous fibrosis (OSMF) in Asia associated to betel nut usage has been extensively characterized both clinically and histologically, the effects of snus (a traditional Scandinavian oral tobacco product) use in Europe are far less well understood. With the growing popularity of snus across Europe, its impact on oral mucosal pathology has become an issue of increasing clinical relevance. In this study, 50 patients were examined, presenting with clinically detectable oral mucosal alterations, associated with habitual snus use. Clinically, the lesions typically appeared as leukoplakic, firm mucosal changes with surface corrugation. In the most severe cases, biopsies were obtained and histopathologically and immunohistochemically analyzed. This evaluation revealed lymphocytic infiltration, epithelial hyperplasia with keratinization, and varying degree of submucosal fibrosis. These findings demonstrate that snus use can induce significant pathohistological manifestations in the oral mucosa, closely analogous to those of the established potentially malignant disorder OSMF. Additional periodontal and dental effects, including gingival recession, erosions, and tooth discoloration, were also recorded. This study provides novel insights into the potential link between snus use and OSMF-like pathology and underscores the importance of vigilant clinical monitoring of affected individuals.

## Introduction

In numerous Asian countries, particularly in India, a well-established association exists between the habitual consumption of betel nut and chewing tobacco and the induction of oral submucous fibrosis (OSMF) [1]. OSMF is a chronic inflammatory disorder characterized by progressive submucosal fibrosis. It is recognized as a potentially malignant disorder of oral squamous cell carcinoma, with reported malignant transformation rates ranging from 2 to 8% [2]. In populations with high exposure, up to 66% of oral carcinomas are attributed to chewing tobacco products [3–5].

While the relationship between betel nut and chewing tobacco use and OSMF in Asia has been extensively studied, potential parallels with snus, a traditional Swedish smokeless tobacco product, remain largely unexplored in Europe [6]. Snus, derived from the Swedish term for “snuff,” is a moist, ground tobacco preparation, typically portioned in sachets containing humectants, salts, and flavoring agents, and is usually kept in the upper labial vestibule. The duration of use generally ranges from 20 to 60 min, although some users keep it in place for several hours. Its popularity is steadily increasing in Europe [7, 8].

Existing international literature, primarily from Scandinavia, has mainly described snus-associated oral changes as localized white corrugated lesions, commonly referred to as smokeless tobacco keratosis, which represents the most frequent clinical finding [9]. Most studies emphasize that these lesions are reversible following the cessation of snus use, with regression typically occurring within weeks to months [10, 11]. Although the carcinogenic potential of smokeless tobacco has been repeatedly highlighted, large Nordic cohort studies have not consistently demonstrated an elevated risk of oral cancer to date [3, 12].

To the best of our knowledge, snus-associated oral lesions have not been described or diagnosed as OSMF, and no prior study has systematically correlated clinical and histopathological findings. Therefore, the present study aimed to address this gap by providing the first systematic clinicopathological correlation of snus-associated oral lesions in a European cohort and contextualizing these findings within the established understanding of OSMF.

## Material and methods

The observational cohort consisted of 50 patients who presented during routine dental consultations between 2022 and 2024 with clinically evident mucosal alterations associated with snus use. The inclusion criteria comprised clinical mucosal changes, clearly attributable to habitual snus consumption.

Standardized documentation included detailed information on snus use (duration, frequency, and average placement time) and subjective symptoms such as pain, burning sensations, or discomfort. A uniform clinical assessment protocol was applied to record characteristics, including localization, size, color, surface texture, and consistency on palpation. Digital photographs were obtained to enable consistent longitudinal comparisons.

In accordance with the German S3 guidelines [13], biopsies were performed in three patients with persistent or progressive lesions. Tissue specimens were fixed in formalin, embedded in paraffin, and stained with hematoxylin and eosin (H&E). Additional immunohistochemical analyses (CD3, Ki-67, and ERG staining) were performed. Histopathological evaluation was performed by a specialized head and neck pathologist (S.I.).

Most cases were managed conservatively through patient education, behavioral counseling, and regular clinical follow up. Patients were enrolled in a structured recall system with control intervals of 3–6 months, during which reexaminations and repeat photographic documentation were performed.

This study was conducted in accordance with the ethical principles of the Declaration of Helsinki (latest revision) and complied with all relevant institutional and national research ethics guidelines.

## Results

Over a 3-year period, 50 male patients with clinically detectable oral mucosal alterations associated with habitual snus use were identified during routine dental examination. None of the individuals presented with symptoms specifically due to these mucosal changes.

The mean duration of snus use was 3.3 years (SD 1.1), with a median daily consumption of three sachets (IQR 2–5). Most participants reported using snus as an alternative to cigarette smoking, although a considerable proportion had no prior history of smoking. Several users had adopted snus as part of their smoking cessation attempts, and younger participants expressed a willingness to discontinue its use in the future.

Clinically, the mucosal alterations appeared as localized, non-wipeable, leukoplakia-like lesions exclusively located in the upper vestibule. The lesions were typically firm on palpation, exhibited surface corrugation or folding, showed varying degrees of discoloration, and were largely asymptomatic. In addition to mucosal alterations, clinical evaluation frequently reveals periodontal and dental changes, most notably gingival recession, cervical defects, and tooth discoloration.

Biopsies were obtained from three patients with persistent or progressive lesions that were clinically suspicious. These three cases represent the histopathologically examined subset of the larger clinical cohort and were selected due to persistence, progression, or clinical suspicion of the lesions. The clinical and histopathological characteristics of these three cases are described in detail in the following sections.

### Case 1:

The patient reported using snus once daily for three years and did not experience any subjective complaints. Clinically, a slightly elevated, nonwipeable, whitish lesion with surface folding was observed in the right vestibule. The lesion was dry, firm, and asymptomatic, with diffuse borders, but no involvement of the attached gingiva or teeth. There were no signs of ulceration.

**Fig. 1:**
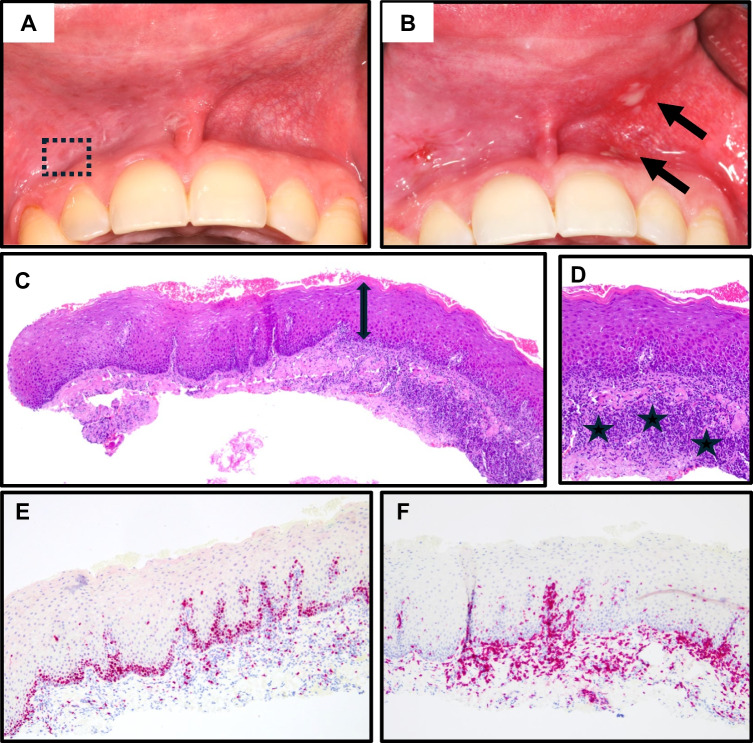
Case 1. **A** Preoperative situation with snus use exclusively on the right vestibular side (dashed rectangle). **B** After 10 days of continuous snus use on the left side during postoperative healing, early mucosal alterations became apparent (arrows). **C** Moderate epithelial thickening (double arrow) with focal parakeratosis. **D** Moderate lymphocytic infiltration (stars) without enhanced fibrosis. **E** Ki67 proliferation within normal range. **F** CD3 staining highlighted moderate numbers of intraepithelial lymphocytes

Histopathological analysis revealed moderate epithelial thickening with focal parakeratosis and minimal spongiosis, without signs of atypia. The corium showed intense lymphocytic infiltration with few plasma cells and no neutrophils. PAS and Ki67 staining were unremarkable, while CD3 staining confirmed moderate intraepithelial T-lymphocyte infiltration (Fig. 1A).

### Case 2:

In contrast to Case 1, this patient reported a longer and more intense history of snus use, averaging three sachets daily for five years, and complained of dental pain with pronounced cold sensitivity. The mucosal alterations were more extensive and clinically more conspicuous, presenting in the left upper vestibule (teeth 21–23) as a whitish-gray, non-removable plaque with pronounced surface wrinkling and hyperkeratosis. Severe gingival recession of the affected teeth was evident, distinguishing this case from the milder findings in case 1.

**Fig. 2:**
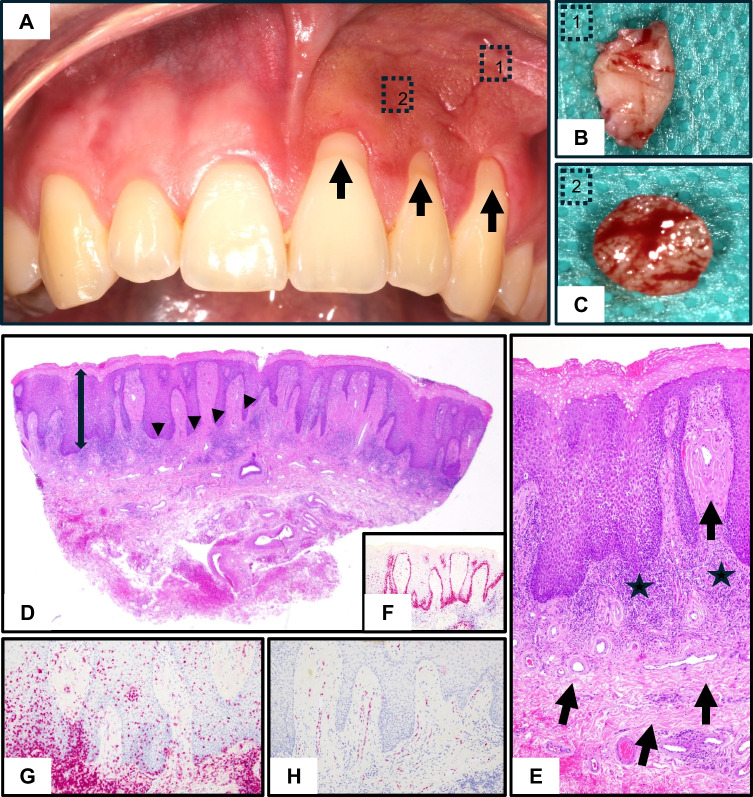
Case 2. **A** Preoperative situation with pronounced gingival recession in the region of teeth 21–23 (arrows). **B**, **C** Biopsy specimens corresponding to the marked areas in (A). D-H Histology of biopsy speciment 2: **D** *Pronounced epithelial thickening (double arrows) with acanthosis and papillomatosis (arrowhead)*. **E** *Dense lymphocytic infiltrate with plasma cells (stars) and focal submucosal fibrosis (arrow)*. **F** *Ki67 proliferation was moderately increased.* **G** CD3 staining highlighted moderate intraepithelial lymphocytes. **H** ERG staining demonstrated reduced capillary density

Histopathological findings were mainly characterized by pronounced epithelial acanthosis, papillomatosis, and parakeratosis. The inflammatory infiltrate was dense, with an abundance of plasma cells and scattered neutrophils. A key finding was additional focal submucosal fibrosis with reduced vascular density, as demonstrated by ERG staining. Notably, biopsies were taken from two different sites (Fig. 2B/C: peripheral/central) showed heterogeneity; one area exhibited more active inflammation with early fibrosis (2B), whereas another demonstrated advanced fibrotic remodeling with less inflammation (2C).

### Case 3:

The patient had been using snus for six years at a rate of four to six sachets per day and reported pain affecting both the oral mucosa (spontaneously and upon palpation) and teeth. Clinically, the lesion extended from region 12 to 22, appeared leathery and indurated. Additional features included marked brownish discoloration, abfractions at teeth 13 and 23, severe gingival recession, and visible tobacco staining.

**Fig. 3:**
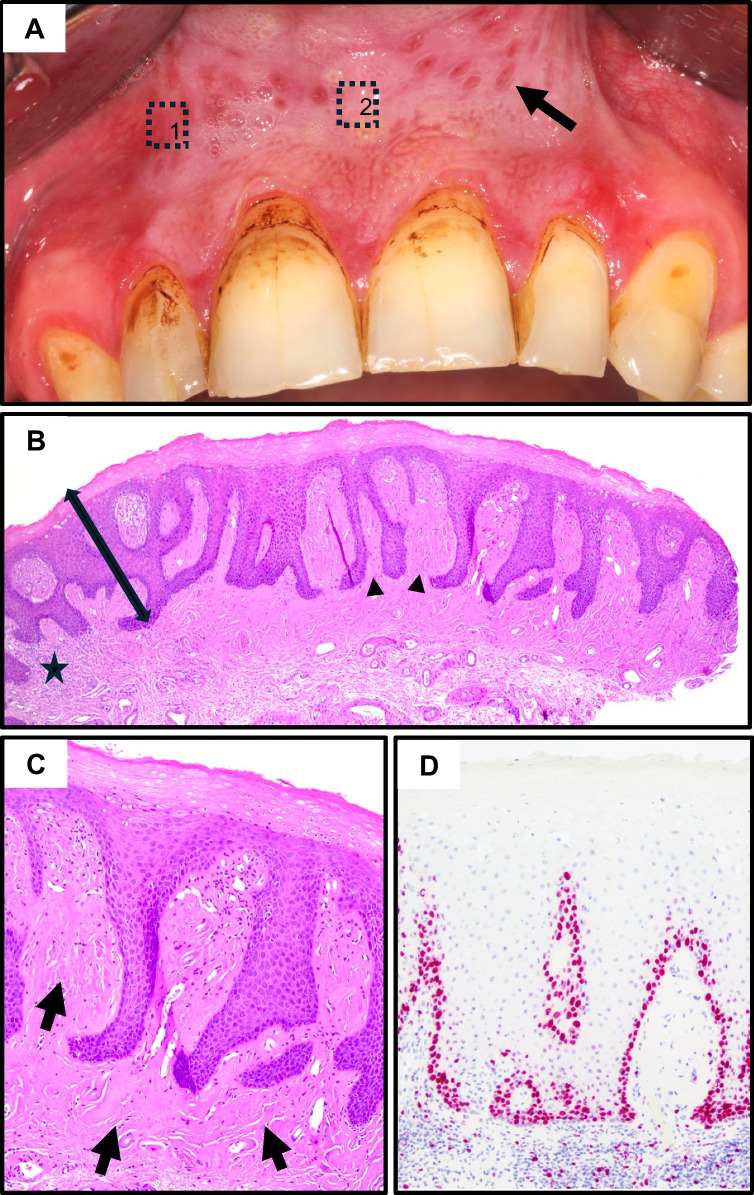
Case 3. **A** Preoperative situation with an extensive mucosal alteration of the upper vestibule; biopsy areas indicated (1,2). **B** Marked epithelial thickening (double arrow) with acanthosis and papillomatosis (arrowhead), minimal spongiosis and minimal lymphocytic infiltration (star). **C** Marked submucosal fibrosis (arrows). **D** Ki67 staining showed mildly increased proliferative activity

The lesion shared the histopathological features of hyperkeratosis, acanthosis, and papillomatosis seen in the cases 1 and 2, but showed widespread submucosal fibrosis with scarce remnants of lymphocytic infiltrates. Ki67 showed a mild increase in proliferative activity, whereas PAS remained negative. Taken together, these three cases indicate presumable progression from early reactive mucositis in Case 1, through initial fibrosis in Case 2, and extensive fibrotic remodeling in Case 3 (Fig. 3).

## Discussion

Oral submucous fibrosis (OSMF) is a chronic inflammatory and fibrotic disorder of the oral mucosa that is highly prevalent in populations exposed to betel nut and tobacco chewing, particularly in India [14, 15]. Classified by the World Health Organization as a potentially malignant disorder, OSMF represents a major etiological factor for oral squamous cell carcinoma in these regions [3, 4]. Its pathogenesis involves arecoline-induced activation of TGF-β1 signaling, disturbed collagen metabolism, and oxidative stress, collectively promoting carcinogenesis [16].

In Europe, this situation is markedly different. Snus, a traditional Swedish smokeless tobacco product, has gained increasing popularity and has been well studied epidemiologically, but clinical and histopathological data remain scarce [10, 11]. This study provides, for the first time in a small European cohort, histopathological evidence linking snus-associated mucosal changes to the diagnostic concept of OSMF.

The findings in 50 patients indicate that snus use typically follows a short but intensive pattern of consumption, particularly among young males who may be at elevated risk due to behavioral and social factors promoting habitual use. Snus may serve either as a primary nicotine source or a substitute during smoking cessation attempts.

The most common clinical finding was the presence of leukoplakic corrugated mucosal lesions, primarily in the upper vestibule. Biopsies were performed in three patients with persistent or progressive lesions. In the patient with limited exposure (Case 1), inflammation predominated, characterized by marked lymphocytic infiltration and mild epithelial thickening. With prolonged and more intensive use (Case 2), focal fibrotic remodeling, acanthosis, and hyperkeratosis were observed, accompanied by reduced inflammation. The most severe case (Case 3) demonstrated extensive submucosal fibrosis with minimal residual inflammation, consistent with the classical histopathological features of a fully developed OSMF. These findings align with the concept that fibrosis increases while inflammation declines in advanced stages [17, 18]. Once established, fibrotic changes are regarded as irreversible in OSMF [19]. Although snus-related lesions have traditionally been considered reversible, the present cases suggest that beyond a certain stage, snus-associated alterations may become irreversible, paralleling OSMF [20, 21].

In contrast to betel nut chewing, snus does not contain arecoline. Fibrotic remodeling in snus users may instead be driven by chronic hypoxia, mechanical irritation, and exposure to tobacco-specific nitrosamines [22, 23]. Evidence regarding carcinogenicity remains inconsistent; several long-term studies have not detected dysplasia or malignancy in biopsies from snus users [24, 25], whereas others have reported potentially malignant changes in up to 6.2% of cases [16]. None of the biopsies in the present series revealed epithelial dysplasia, possibly reflecting the relatively short exposure duration and the limited sample size. Although current data do not support classification of snus-associated lesions as a potentially malignant disorder, the histological similarities to OSMF warrant caution and emphasize the need for continued clinical surveillance.

Even though snus-associated lesions so far are not classified as potentially malignant disorders, snus use causes clinically significant harm to periodontal tissues and teeth. Chronic hypoxia, mechanical irritation, and exposure to tobacco-specific nitrosamines contribute to vasoconstrictive and proinflammatory mechanisms that impair periodontal tissue response and delay healing after trauma or surgery. Documented clinical findings, as observed in the present cases, include abfractions, abrasions, and tooth discoloration [26, 27]. Gingival recession has been reported in up to 17.8% of users, particularly among those consuming loose snus [28]. These periodontal and dental changes are irreversible, even though fibrotic remodeling of the mucosa may become irreversible only after a certain stage of fibrosis is reached. Moreover, epidemiological studies indicate that snus users have an approximately two-thirds higher risk of tooth loss than non-users, although this association may be influenced by oral hygiene and lifestyle factors [29]. An inverse relationship was observed between oral hygiene and the extent of snus use, consistent with reports linking snus to periodontitis progression [30]. Painful exposure of tooth roots may lead to neglected oral hygiene, further aggravating periodontal destruction. Notably, many users continued consumption despite these issues, underscoring the addictive potential of snus.

In summary**,** the findings suggest that snus-associated lesions in Europe are histologically comparable to the early stages of OSMF, although without evidence of dysplasia or carcinoma. While this may offer tentative reassurance, the observation periods remain too short for firm conclusions, and the limited cohort size restricts generalization. Continued clinical monitoring, longitudinal studies, and individualized risk evaluations are essential. While snus-associated mucosal fibrosis could become irreversible beyond a certain stage, the accompanying periodontal and dental damage seems to be permanent. Although malignant transformation has not yet been demonstrated, the clinical consequences of snus use are substantial and require careful monitoring. These findings underscore the importance of preventive strategies, patient education, and a structured follow-up. Regular six-month recall intervals are recommended to ensure the early detection of progression or complications and to support cessation efforts in affected individuals.
